# At What Age Teeth Should Have the Most Attention

**Published:** 1885-03

**Authors:** Geo. A. Mills

**Affiliations:** Brooklyn.


ARTICLE IV.

### AT WHAT AGE TEETH SHOULD HAVE THE MOST ATTENTION.

BY DR. GEO. A. MILLS, BROOKLYN.

I regard the time between the ages of fifteen and twenty-one, particularly in young ladies, as the most critical period we have to deal with. Young girls passing through the age of puberty are in an extremely sensitive condition of body and mind, and that has a great deal to do with directing, and causing divergence in all the operations of growth and repair. It is a period in which we should be very careful and work understandingly, for we can be the means of doing much mischief. I speak more particularly of young girls. Boys are bad enough. The whole matter of the treatment of children's teeth is something we ought to be patient about and discuss fairly, for on it lies the ultimate success of our practice. If it is admitted that every divergence from nature's laws causes irregularity, disturbance and harm, should we extract the temporary teeth? The careless extraction of the temporary teeth, as well as of the permanent, is doing great harm. I question somewhat the idea of feeding the teeth. There may be something in it that I do not understand. I am greatly averse to the feeding of meat to children. Particularly with the American people, I think we are feeding too much animal food. I am acquainted with several persons who are vegetarians in the extreme sense of the word. I often notice these persons, and I find they are what we call conservative men, and that they have a higher standard of organization. But if you are going back to follow out nature's laws, you must go clear behind the growing child into the germinal matter. The sexual relation is a large and important question. The most eminent neurologists in this country are settling down on one general principle, that all diseases have their origin in nervous degeneracy. We must go back and look this thing in the face. The time is coming, perhaps, when those who come after us will understand this better than we are able to do. One thing in this connection is settled,— more highly organized beings are subject to a greater degree of deflection from outward circumstances than lower organizations. We know that in this country, there is a high state of nervous organization among the people, that there is all about us, and in all circumstances, a high-pressure condition largely due to nervous causes. Now, a very large proportion of the children born are generated under these conditions of nervous deflection. Take the germinal matter, the spermatoza, in a highly nervous organization, and we know very well that this germical matter is very far from being in a normal condition. It is subject to the pressure of its atmosphere, and the condition, of this germinal matter and the circumstances under which the child is generated have very much to do with the condition of its mind and body in the future. A great many people in the community are little more than bundles of nerves; and their trouble commenced nine months before they were born. There are marked instances on record of difference in children who were generated by the same parents under different circumstances. It sometimes occurs that the first child is the best child, and sometimes the last child child is the best child.

Now, the reason some are better than the others is that they were conceived under more favorable circumstances. When the progenitors are first married is one of the happiest and most favorable periods for generating well-organized children; they are under new relations of life, generally in good health, filled with all kinds of hopefulness, everything is lovely, and children conceived under such circumstances are likely to be well organized. But go farther along in life and the time comes when there is an adverse change in the circumstances of the parents, financial embarrassment, excessive toil, and many cares harass them, and the children then generated reflect the change in circumstances. Children generated during times of financial crisis, when there is commercial disturbance from top to bottom of society, with sudden loss of wealth and comforts, show a marked deflection from the normal condition, due to a like condition of the germinal matter in their progenitors, caused by sympathy with their changed circumstances. It is falling of the mercury, and the children, when born, are absolutely certain to evidence it.

Consider the man who is not married till he is forty years of age, and then begets children. This man is in a state of maturity, and the children generated under such circumstances are very often of an improved standard of organization. I remember a statement I heard a woman make once, which I thought rather remarkable, as coming from a woman, that when we get to the point were children are properly generated, they will never need any regeneration. The meaning of this is, that when we understand nature's laws and conform to them, when everything is working in harmony, then we get a high state of organization, spiritual as well as animal. Nothing can be truer than this. We are passing through a transition state; the animal and the spiritual in us are struggling with each other.

Go back to the pre-historic ages, when the race lived more on the animal side,—more animal and less spiritual,—and you see, as shown in the skulls belonging to that period which are preserved in our museums, a firstclass type of teeth.

In the skulls of the Romans you see a very perfect development of teeth. Then there was a deflection from this standard, and you see in the skulls of those ages the types of all the dental diseases which we see to-day—caries, necrosis, alveolar abscess, etc. We must be patient, striving for what is possible, not for what we would like to be, and encouraging the youngmen who are coming to take our places. As we get on ahigher plane of civilization, where nature's laws will be understood and observed, we shall more clearly see that prevention is better than cure.—*N. J. Dental Society*.

